# The Intentions to Wear Face Masks and the Differences in Preventive Behaviors between Urban and Rural Areas during COVID-19: An Analysis Based on the Technology Acceptance Model

**DOI:** 10.3390/ijerph18199988

**Published:** 2021-09-23

**Authors:** Bo Zhang, Zhongjie Li, Lei Jiang

**Affiliations:** 1 School of Tourism Management, South China Normal University, Guangzhou 510631, China; zhangborug@m.scnu.edu.cn (B.Z.); 20171131024@m.scnu.edu.cn (Z.L.); 2 Southern Marine Science and Engineering Guangdong Laboratory, Guangzhou 519085, China; 3 School of Economics, Zhejiang University of Finance and Economics, Hangzhou 310018, China

**Keywords:** technology acceptance model, behavioral intention, attitudes, mask wearing, COVID-19 pandemic

## Abstract

The SARS-CoV-2 virus first emerged in late 2019 and has since spread quickly throughout China and become a global pandemic. As the situation with COVID-19 has evolved, wearing a face mask in public has grown commonplace. Using the technology acceptance model (TAM) as a foundation, this study introduces three new variables, namely, perceived risk, social pressure, and social image, to establish an extended model for investigating the factors that influence if residents wear masks. A total of 1200 questionnaires were distributed in China, from 1 February to 30 May 2020, through China’s largest online platform. The results indicate the following: 1. Residents’ positive attitude towards mask wearing promotes their behavioral intention to wear masks. 2. Perceived risk, social pressure, and social image have a positive impact on attitude towards mask wearing. 3. The intention to wear masks and attitude were both positively influenced by perceived usefulness. 4. The perceived usefulness is more influential in rural than urban groups, in terms of behavioral intention. This article proposes that public education on the facts related to the coronavirus, the threats posed by the COVID-19 pandemic to health, and the usefulness of face masks in preventing the transmission of COVID-19 could increase residents’ intention to wear a mask.

## 1. Introduction

Since the end of 2019, the long dark shadow of coronavirus has been cast over people around the world, due to both the COVID-19 outbreak and the rapidly increasing number of cases. More than 130 million cases have been confirmed, and more than 2.8 million deaths have been reported globally since the World Health Organization (WHO) declared COVID-19 a pandemic [1]. The global spread of COVID-19 has seriously harmed the world economy, while also significantly altering humanity’s way of life. To prevent the transmission of COVID-19, the National Health Commission (NHC) issued an announcement on 20 January 2020 that indicated that the SARS-CoV-2 virus was being incorporated into ‘The Law of the People’s Republic of China on the Prevention and Treatment of Infectious Diseases’ and was identified as a Category B infectious disease. Like most other countries, in the document issued, the NHC put forward a series of particular guidance measures such as the disinfection of touched surfaces and COVID-waste, dilution ventilation, and launching a vaccine research and development program. For the general public, social distancing and mask wearing are considered as two significant and relatively simple means to reduce the probability of human-to-human contagion. Face masks, as a type of personal protective equipment (PPE) [2], were widely advised in China in the early stages of the outbreak and became increasingly recommended to stop the transmission of COVID-19 among the general public [3]. The WHO then advocated for the universal use of face masks in epidemic prevention, as a means of personal protection and source control; for healthy people, wearing masks can protect them by allowing fewer coronavirus particles to be inhaled when in close contact with an infected person, while for infected people, the use of masks can prevent onward transmission. According to a study by Hong Kong researchers, Cheng et al. (2020) [4], public mask wearing is effective in reducing COVID-19 transmission in the community, thus curbing the spread of the pandemic. Slowing the spread of the virus by mask wearing can also be applied to the United States, where COVID-19 has widely taken hold [5]. However, a study by Dave et al. (2020) [6] revealed a relatively low rise in COVID-19 cases during the ‘Black Lives Matter’ rallies across the United States, since most protesters wore masks, washed their hands, and took other precautions. With the ongoing emphasis on the preventive function of face masks and the dissemination of relevant knowledge, wearing masks to combat COVID-19 has become a worldwide consensus, and many nations have mandated the use of masks or face coverings in public.

In March 2020, the Chinese government released guidelines and requirements for wearing masks in public settings [7]. Later, a mask mandate was enforced nationwide by governments and administrations at all levels. In particular, the mask mandate was applied in densely populated areas, such as public parks, or confined public places, such as supermarkets, and functioned in cooperation with universal temperature monitoring and social distancing in epidemic areas. Indeed, since the Chinese government issued an administrative order requiring the mandatory usage of masks, the rate of COVID-related infections has been progressively declining, according to statistical data. Given the multi-dimensional analysis of outbreak prevention and control measures, we cannot simply correlate the use of masks with the prevalence of COVID-19 infections. Face masks have been proven in certain studies to be beneficial in reducing the spread of the coronavirus and containing outbreaks [8]. In fact, the Chinese public has generally agreed on the importance of face masks in preventing the spread of the virus, and most are willing to wear mask as a collective action in public. However, research on willingness to mask or not to mask is still scarce. Studies from China have also revealed [9] that some people go unmasked outdoors and even refuse to wear them while in hospitals or clinical premises, thus implying that attitudes and behavioral intentions to wear masks may differ among individuals. To address the notion of willingness, this paper performed an in-depth study on the mask wearing behavior of Chinese residents during the pandemic and made comparisons between urban and rural areas in order to investigate which factors influence mask use.

Based on the theory of reasoned action (TRA), Davis (1985) [10] proposed the technology acceptance model (TAM), which was initially used to explain acceptance of technology by individuals [11], but was then extended to a broader range of studies in the field of technology acceptance behavior [12,13]. If wearing masks is considered to be a protective technology against viral infections, then the TAM can provide a concise and straightforward way to study the acceptance of this behavior. Differently from using demographic characteristics as factors influencing the wearing of face masks, this study takes an extended TAM as the foundation for exploring the behavioral intention to wear masks among Chinese urban and rural residents, employing four dimensions: (1) perceived usefulness, (2) perceived risk, (3) social pressure, and (4) social image, to better understand the motivations influencing mask wearing behavior. From a policy standpoint, this paper also suggests ways to promote greater proactive use of masks by the public.

## 2. Literature Review and Research Hypotheses

### 2.1. Research Model

The technology acceptance model (TAM) was applied as the basic model in this study. TAM comprises two primary dimensions, namely perceived usefulness and perceived ease of use as the external variables. The fundamental meaning of this model is encompassed in three aspects: the attitude of individuals towards technology is impacted by perceived usefulness and perceived ease of use; behavioral intention is influenced by attitude, and individual behavior to embrace technology is ultimately affected by behavioral intention.

‘Perceived ease of use’ is defined as the degree to which a prospective user expects a target system in his or her job to be effortless, meaning that the easier a system is, the more confident users will be in using it correctly and executing its functions properly [14]. It is considered that, in TAM, perceived ease of use has a significantly positive effect on attitudes; namely, the simpler a technology, the more positive the user’s attitude towards using it. However, there is not necessarily a correlation between these two variables. For instance, Lee et al. (2019) [15] reached a similar conclusion in a survey carried out in South Korea on the use of virtual reality (VR) devices, and ascribed this occurrence to customers finding it easy to use VR devices. Similarly, Ooi and Tan (2016) [16] also found that, in an electronic payment system based on near field communication (NFC) technology, the perceived ease of use had no discernible impact on users’ willingness to use. This might be because NFC-based electronic payment operation is less complicated and easier to use than traditional electronic payments, which makes people consider the technology extremely straightforward. According to the findings of the preceding research, even if technology becomes less complicated, individuals will not necessarily increase their propensity to use it. Thus, there is no significant relationship between perceived ease of use and user attitudes towards using when the perceived ease of use is below a particular threshold, e.g., when users find it simple to use, without trouble.

Clearly, the difficulty of using a mask is far lower than that of the technologies employed in the research above, so the correlational relationship between perceived ease of use and attitude towards mask wearing is assumed not to be significant. The WHO issued an interim document in the early stages of the COVID-19 outbreak, instructing the public on how to properly wear a protective face covering: place mask over both nose and mouth, slip the loops over the ears, and ensure a snug fit against the face [17]. A study conducted by Gunasekaran et al. (2020) after the pandemic outbreak, showed that 92.3% of 3261 respondents using masks can wear them correctly [18]. In other words, it is quite simple for the vast majority of people to correctly wear a mask.

**Figure 1:**
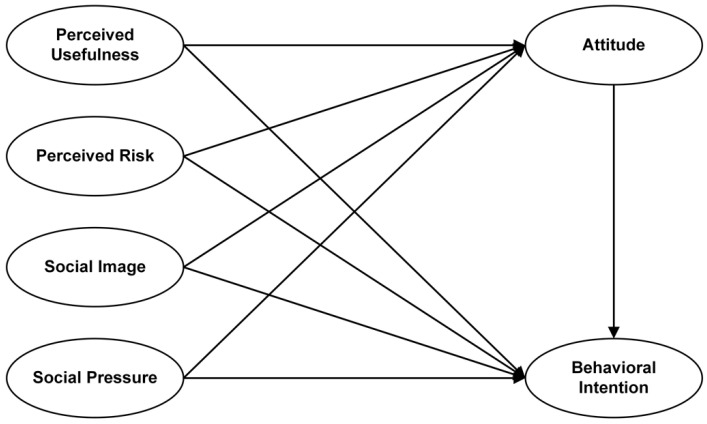
Research Model.

In fact, perceived usefulness and perceived ease of use are also affected by external variables. However, the explanation of external variables is not always required in the TAM model, thus signifying that the TAM is an open model and may be flexibly used by researchers based on the given circumstances. Indeed, there is robust evidence indicating that pandemics have a strong impact on people’s behavioral intentions [19,20,21]. Protective behavior in particular is usually promoted by the perceived risk [22], which is considered as a significant factor influencing people’s intention to adopt self-protective behaviors, including social distancing and face mask wearing during pandemics [23,24]. As a result, people’s risk perception of COVID-19 may be one of the variables influencing the use of face masks. In addition, a recent study showed that concerns about peer-pressure could affect individual mask wearing behavior [25]; the implication here is that social environmental factors such as social image and social pressures should also be considered. Hence, based on the original TAM model, the three variables of perceived risk, social image, and social pressure were added to finalize our research model, as shown in Figure 1.

### 2.2. Research Hypotheses

#### 2.2.1. Attitude and Behavioral Intention

Attitude can be described as the tendency to react favorably or adversely to a person or set of circumstances, whereas behavioral intention is the probability or strength of the intention to follow a particular behavior [26]. The theory of planned behavior (TPB) [27], as well as the technology acceptance model (Davis, 1985) [10], which was developed out of TPB, argue that attitude is an important prerequisite for behavioral intention. In fact, numerous empirical research reports show that the relationship between attitude and behavioral intention is stable and substantial [11,12,13]. Therefore, in terms of mask wearing behavior, the more positive people’s attitudes are towards the use of masks, the greater is their behavioral intention to wear them.

A hypothesis is presented below based on the above points of view:

**Hypothesis** **1** **(H1).** *Residents’ attitudes towards masking have a significant and positive effect on their behavioral intentions to wear masks or face coverings*.

#### 2.2.2. Perceived Usefulness

According to Davis et al. (1989) [14], the definition of perceived usefulness refers to a person’s subjective perception that using a particular system will enhance his or her performance. In other words, the prospective users are more likely to adopt a specific application system when they believe it will help them attain gains in a certain aspect. In this study, perceived usefulness is interpreted as respondents’ perceptions that wearing a mask has its own advantages as a means of personal protection during the COVID-19 outbreak, i.e., people tend to wear masks when they feel that masks provide indisputable protective effects.

Individuals’ attitudes and behavioral intentions towards technology are substantially affected by perceived usefulness in the TAM model, the correlation of which has been consistently verified by many researchers. For example, a study on college students’ acceptance behavior of online learning technologies that integrate multiple online resources indicated that perceived usefulness is significantly correlated with college student attitudes and intentions to use online learning technologies [28]. Another study on student behavioral use of tablet computers noted that perceived usefulness significantly and positively influenced their attitudes and intentions with regard to tablet usage [29].

The two hypotheses below are based on the aforementioned perspectives:

**Hypothesis** **2** **(H2).** *Perceived usefulness has a significant and positive effect on residents’(a) attitudes towards mask-wearing and (b) behavioral intentions to wear masks*.

#### 2.2.3. Perceived Risk

Perceived risk, also known as perceived likelihood and perceived vulnerability [30], is the perception of the severity of a particular health threat and the likelihood of actual harm being caused by that threat [31]. It is generally accepted that risk perception is a significant theme in health behavior theories, such as the protection motivation theory. In this study, perceived risk is defined as the public’s perception of the severity of catching COVID-19 and the probability of getting infected with this virus; and perceived risk leads individuals to engage in self-protective behaviors to combat the disease [32]. For example, Gong et al. (2020) demonstrated that perceived risk positively and significantly impacted on the adoption of self-protective measures in an influenza protection study [33]; De Bruin and Bennett (2020) found that people are more likely to adopt self-protective measures to prevent coronavirus if they believe that COVID-19 is seriously harmful to the human body and that they may be at high risk of infection [34]. However, differently from a typical virus, the asymptomatic transmission of COVID-19 prevents individuals from determining if they and others potentially carry the virus, and this unknowable factor may heighten their concern about the pandemic [35]. Based on the above studies, we can hypothesize that the higher the risk of catching COVID-19 when in public, and the more serious the consequences are of infection, the more likely it will be that individuals will wear masks.

Two hypotheses are proposed below based on the perspectives discussed above:

**Hypothesis** **3** **(H3).** *Perceived risk contributes a significant and positive impact on people’s(a) attitudes towards mask wearing and (b) intentions to wear masks*.

#### 2.2.4. Social Image and Social Pressure

People care about how they are perceived by others, and social image concerns seem to influence a wide range of behaviors [36]. ‘Image’ refers to the degree to which the use of an innovation is seen to improve one’s social status [37]. Davis (1993) found that image played a primary role in the specific example of mobile phone adoption or use [11]. ‘Social pressure’, on the other hand, is described as a kind of value or behavioral pattern that holds that individuals should abide by the rules and practices accepted by the general public [11]. Wearing a mask, in fact, may encourage the wearer and people around them to adhere to other rules, such as keeping social distance [38]. Other scholars have proven that social pressure exerts an influence on the use of technology [39]. For instance, Kaba et al. (2009) discovered that social pressure has a substantial impact on attitudes towards cell phone use, which is influenced by the frequency of phone calls [40]. Moreover, Anandarajan et al. (2002) have shown unequivocally that social pressure is the main cause for the adoption of technology in a collaborative culture [41].

Hofstede (1994) proposed that in a collectivist society, people integrate themselves into the collective, believing that the collective can protect them when staying loyal to it [42]. Due to a high level of trust among collective members, individual behavior is impacted by the collective. China is a country that emphasizes collectivism, and Chinese culture has long put a greater emphasis and concern on the general interests of society [43]. The act of wearing a mask helps to prevent and control outbreaks by reducing the risk of virus transmission and ultimately leads to the protection of the general interests of society. The State Council of China published a ‘Notice on Issuing Guidelines for Scientific Wearing of Masks by the Public’ in March 2020, highlighting the need to wear masks in crowded public settings [44].

The four hypotheses proposed below are based on the aforementioned perspectives:

**Hypothesis** **4** **(H4).** *Social image has a significant and positive impact on people’s (a) attitude towards mask-wearing and (b) behavioral intention to wear masks*.

**Hypothesis** **5** **(H5).** *Social pressure has a significant and positive impact on people’s (a) attitude towards mask-wearing and (b) behavioral intention to wear masks*.

#### 2.2.5. A Comparison of Urban and Rural Residents

There are health disparities between rural and urban residents in terms of health care resources (e.g., transportation, health insurance, providers, and facilities). Geographic distance and lower socioeconomic status potentially contribute to higher mortality rates from infectious diseases and pandemics such as COVID-19 [45,46,47,48]. Furthermore, several studies on urban and rural behavior found differences in technology use between urban and rural residents. Eiksund (2009) pointed out that the impact of attitudes towards road safety on risky behavior may differ between urban and rural young drivers [49]; and Porto et al. (2017) showed that the acceptance and use of credit cards differ substantially between rural and urban residents [50]. In our context, masks are recognized as a preventative health resource, but their use tends to vary geographically. For example, in many Asian countries such as China, Japan, and Korea it has been fairly common to wear masks during the pandemic, mostly because masks are considered as a hygienic practice in these countries. However, this perception contrasts quite dramatically between East and West, with many Western countries regarding mask wearing as a violation of freedom and individualism [51]. Given our discussion thus far on masks, it is apparent that attitudes and behaviors are influenced by the perceptions held by people in different regions. Hence, there may also be variations between urban and rural areas when it comes to the use of face masks, which is understood not only as a relatively simple technology, but also as a preventive health behavior by a healthy individual keen to avoid disease. Data from the Pew Research Center suggests that rural communities have a larger percentage of conservative-leaning voters, who are more resistant to wearing masks [52].

The following hypotheses are proposed based on the preceding discussion and the attending views:

**Hypothesis** **6** **(H6).** *There is a significant difference between urban and rural residents in the influence of (a) perceived usefulness, (b) perceived risk, (c) social image, and (d) social pressure on the attitude towards mask wearing*.

**Hypothesis** **7** **(H7).** *There is a significant difference between urban and rural residents in the influence of (a) perceived usefulness, (b) perceived risk, (c) social image, and (d) social pressure on the behavioral intention to wear masks*.

**Hypothesis** **8** **(H8).** *There is a significant difference between urban and rural residents in the influence of attitude towards mask wearing on behavioral intention*.

## 3. Data and Method

### 3.1. Data Source

We adopted a questionnaire survey to collect data for this study, and the research scale was designed by consulting a number of scholarly articles, e.g., Davis (1985) [10], Moore and Benbasat (1991) [37], Karahanna et al. (1999) [53], Fishbein and Ajzen (1977; 1985) [26,27], and Kwon and Chidambaram (2000) [54]. The final questionnaire was prepared after revisions and improvements were made with the help of scholars and professionals in the field. In the pilot, twenty (20) respondents were then chosen at random to fill out the questionnaire prior to being distributed online, and the relevant statements and expressions were further clarified to finalize the measurement scale, based on solicited feedback. All of the questions were asked on a 7-point Likert scale (7 = ‘extremely consistent’, and 1 = ‘not at all consistent’) to measure respondent opinion or attitude towards a specific issue. The questionnaire was divided into two (2) sections, with urban and rural residents across China serving as research subjects. Specifically, Part A consists of measuring research variables and Part B comprises a survey of demographic characteristics, including gender, age, educational attainment, and monthly income. From 1 February to 30 May in 2020, it was administered via Questionnaire Star (Wenjuanxing in ChineseChina’s largest online questionnaire platform with 16.39 million active users in various demographic characteristics. All participants had been living in China during the COVID-19 pandemic and are literate in Chinese. A random sampling method was conducted through Questionnaire Star, yielding the distribution of a total 1200 questionnaires. Of these, 1144 were returned, giving a response rate of 95.33%.

### 3.2. Method and Statistical Analysis

The analysis was performed using SPSS Statistics 22.0, and results are presented in Table 1. Of those who responded, 35.1% were men and 64.9% were women. As can be seen, females outnumbered males in the sample size. The age range spanned a wide spectrum (e.g., under 18 and over 50). Respondents aged 19 to 29 accounted for the majority of the total (44.5%), while those aged 18 and younger comprised the smallest proportion (4.4%). The education attained by survey respondents encompassed all levels of school credentials, from lower to upper tiers, with more than half (53.5%) receiving a bachelor’s degree and only a small percentage (1.7%) ending in lower secondary and below schooling. On the income side, those earning 5000 yuan or less (equivalent to EUR 653 and USD 774) a month contributed 53.8%, followed by those with an average monthly income of 5001–10,000 yuan (31.4%), and high earners with 15,001 yuan or more represented the lowest proportion, at just 5.4%.

## 4. Results Analysis

### 4.1. Reliability and Validity Test

Confirmatory factor analysis (CFA) was performed to verify whether the observed data suited the model well, with the results set out in Table 2. We verified that the entire model fit indices (CFI = 0.948; GFI = 0.909; NFI = 0.940; SRMR = 0.056; RMSEA = 0.072) met the minimal fit criteria (CFI > 0.900; GFI > 0.900; NFI > 0.900; SRMR < 0.050; RMSEA < 0.080). The predicted model therefore corresponds closely to the observed facts. In this study, the standardized loadings between all measured items and variables ranged from 0.624 to 0.955, and therefore fall within the reasonable scale between 0.500 and 0.950. The lowest composite reliability (CR) and average variance extracted (AVE) were 0.762 and 0.519, respectively, which is higher than the recommended value of composite reliability (CR) >0.600 and average variance extracted (AVE) >0.500. In addition, the lowest Cronbach’s α value was 0.756, which exceeds the minimal requirement 0.70. All these numbers indicate that the model has strong reliability and validity [55,56].

### 4.2. Model Comparison

To test the validity of the model, a confirmatory factor analysis was performed, as showed in Table 3. Five competitive models were proposed and evaluated to determine the best one using the data. Results are presented in Table 3. Significant variations in Chi-square values (∆χ2) were found across the four sets of models (Model 1 vs. Model 2; Model 2 vs. Model 3; Model 3 vs. Model 4; and Model 4 vs. Model 5), indicating that the null hypothesis is rejected (the null hypothesis is that Model 2 is a better fit than model 1, and by parity of reasoning as Model 3 vs. Model 2, Model 4 vs. Model 3, Model 5 vs. Model 4). The six-factor model (Model 1: χ2 = 942.748; df = 136; GFI = 0.909; CFI = 0.948; RMSEA = 0.072), consisting of ‘perceived usefulness’, ‘perceived risk’, ‘social image’, ‘social pressure’, ‘attitude’, and ‘behavioral intention’ was the only model to meet the minimal fit criteria (GFI > 0.90; CFI > 0.90; RMSEA < 0.10), thus indicating that, of those proposed, Model 1 is the most stable. Therefore, a six-factor model was employed in the study that followed.

### 4.3. Model Checking

**Figure 2:**
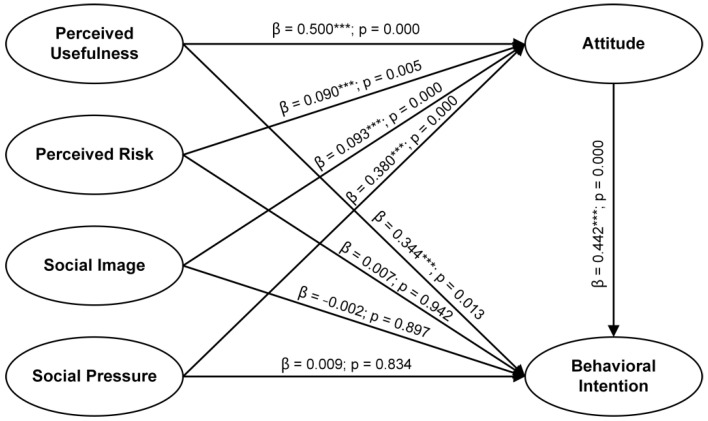
Model Checking Results. Note: Model Fit: CFI = 0.948; GFI = 0.909; NFI = 0.940; SRMR = 0.056; RMSEA = 0.072. ***: *p* < 0.001.

Figure 2 illustrates the results of the structural equation model analysis. First, the variables ‘perceived usefulness’ (β = 0.500; *p* = 0.000), ‘perceived risk’ (β = 0.090; *p* = 0.005), ‘social image’ (β = 0.093; *p* = 0.000), and ‘social pressure’ (β = 0.380; *p* = 0.000) had a substantial and positive impact on attitude, H2a, H3a, H4a, and H5a and were thus supported. Second, attitude significantly and positively affected the behavioral intention and thus H1 was supported. Next, ‘perceived usefulness’ influenced the behavioral intention considerably and positively, so H2b was supported. Lastly, ‘perceived risk’ (β = 0.007; *p* = 0.942), ‘social image’ (β = −0.002; *p* = 0.897), and ‘social pressure’ (β = 0.009; *p* = 0.834) had no significant effect on behavioral intention, thus H3b, H4b, and H5b were rejected. The results of hypothesis testing are presented in Table 4.

### 4.4. Group Difference Test

#### 4.4.1. Model Identity Test

Before carrying out the model identity analysis, the sample dates were divided into two groups according to type of place of residence; namely, the urban group (*n* = 899) and the rural group (*n* = 245). The differences between the non-restricted and full-metric invariance models were analyzed, with the restrictions that in the full-metric invariance model, factor loadings, factor variances, and path regression coefficients were equivalent. The results of the analysis are shown in Table 5. The differences in Chi-square values (∆χ2) between the non-restricted and full-metric invariance models were not significant (∆χ2 (8) = 18.564; *p* = 0.137), indicating no significant difference between the models, that the grouping based on type of place of residence does not affect the applicability of the models, and that the measured models are invariant and can be used for multi-group analysis.

#### 4.4.2. Multi-Group Analysis

The full-metric invariance model was applied to test the differences in group paths between the urban and rural group samples. Results of the multi-group analysis are shown in Table 6. First, the effects of perceived usefulness, perceived risk, social image, and social pressure on attitude were not significantly differentiated between the urban and rural groups; H6a, H6b, H6c, and H6d were thus rejected. Second, in terms of the influence of attitude on behavioral intention, no significant variation between the urban and rural groups was identified; H8 was therefore rejected. Finally, there were no differences in the effects of perceived risk, social image, and social pressure on behavioral intention between the urban and rural groups, but a significant difference in the effect of perceived usefulness on behavioral intention (∆χ2 (1) = 4.411; *p* = 0.036 < 0.05); and therefore H7b, H7c, and H7d were rejected. The analysis presented above reveals that the perceived usefulness of masks yielded a bigger impact on rural residents’ intention to wear masks (β = 0.587; *p* < 0.001) than on urban residents (β = 0.300; *p* < 0.001).

## 5. Concluding Remarks and Policy Implications

The effectiveness of mask use in preventing the transmission of COVID-19 has been adequately addressed since the outbreak of the pandemic [57]. This study analyzed mask use as a personal protection technique on the basis of the technology acceptance model (TAM) during the COVID-19 pandemic. In general, the hypothesis testing showed a significant relationship between attitude and behavioral intention; that is, if residents hold positive attitudes towards using masks, they are more likely to perform mask wearing behavior. Meanwhile, three additional variables—perceived risk, social image, and social pressure—were added to construct an extended TAM. The results demonstrated significant relationships between the extended variables (perceived risk, social image, and social pressure) and attitudes. This means that, although perceived risk, social image, and social pressure do not have a direct impact on people’s behavioral intentions, they do have an impact on people’s behavioral intention through an important mediating variable, attitude. A multi-group analysis was applied in the final stage to test the group difference between rural and urban residents in terms of mask use behavior. Therefore, from a theoretical perspective, it is reasonable to conclude that TAM can be applied, not only to information technology research, but also to the study of mask use behavior.

The key findings are summarized as follows: First, as hypothesized, perceived usefulness is positively related to both the attitudes and behavioral intentions of the residents. In other words, individuals are more inclined to use masks when they believe that wearing masks would provide them with some kind of protection. This coincides with Amoako-Gyampah’s (2007) finding that the use of the technology (if we see mask wearing as a special and easily used technology) is influenced by the perceived usefulness. In other words, increasing the perceived usefulness will lead to a positive intention to wear masks for the general public [58]. In March 2020, an online survey about COVID-19 knowledge and mask wearing behavior was conducted via the Chinese Center for Disease Control and Prevention’s official WeChat account. It collected 5761 questionnaires from 31 provinces, municipalities, and autonomous regions of mainland China [25]. According to the survey, prior to the COVID-19 outbreak, 20.1% of respondents never wore a mask, even though they had developed respiratory symptoms, and 41.1% did not wear a mask while in hospital or other health care settings. The use of masks, however, has changed greatly since the pandemic began; the percentage of individuals unmasked in hospitals and clinics has plummeted to 0.1%, and only 0.1% are unmasked while out shopping. The survey also indicated that the extensive publicity by governments and medical institutions on the usefulness of masking is one of the causes of the growth in the number of individuals wearing masks; boosting public knowledge that masking may in fact positively help prevent COVID-19. Thus, we can observe that residents are more willing to wear masks when they learn that they can offer better personal protection to wearers during the pandemic.

Second, perceived risk has positively affected attitudes towards mask use, but has had no substantial effect on behavioral intention to wear masks. The implication here is that the higher the perceived risk of catching COVID-19 and the more serious the consequences of infection, the more positive are residents’ attitudes towards face masks, which, therefore, will finally lead to individuals wearing masks. However, the recent research by Dryhurst et al. (2020) associated risk perception directly with the protective behavior [23]. In fact, the health belief model (HBM) also claims that individuals’ risk perception is positively and directly related to the protective behavior [59]; however, this article argues that the risk perception promotes the behavioral intention through a significant mediator, attitude, which is in conformity with Bae and Chang’s (2020) findings in South Korea [60]. This means that the authorities would have to change residents’ attitudes towards mask wearing by explaining the threats that COVID-19 causes to residents’ health; and in doing so, promoting the behavioral intention to wear masks. In particular, media coverage of the harm of the coronavirus may contribute to the knowledge of residents, so that a positive attitude towards face masks can be developed, and in turn increase the intention for the protective behaviors.

Third, similarly to perceived risks, social image and social pressure were found to be positively correlated with mask wearing attitudes, but not with the behavioral intention to wear masks. People have positive attitudes towards masks when they feel that wearing one can improve their social standing or when society as a whole believes that individuals should wear masks, but the mask wearing behavior does not materialize immediately. As a result, it cannot be deduced that wearing masks during the pandemic is an attitude that conforms to the social values of the general population rather than just a simple behavior. In fact, mask wearing has been advertised by local, as well as central, authorities in China during the pandemic as, not only a means of self-protection, but also as the social rule and practice to respect lives and the collective work of people against COVID-19. Therefore, under such social pressure and social norms, individuals would consciously hold a positive attitude towards wearing masks when out in public. To reiterate, it may be a good idea for the authorities to change attitudes through public education rather than simply issuing mandatory executive orders if they want to promote mask wearing behavior more effectively. Specifically, publication of the usefulness of face masks, the risk of transmission of COVID-19, and the social influence of wearing masks to protect against epidemics is significant and relevant.

Last but not least, the results of the multi-group analysis indicated that residents from rural areas have a stronger influence of perceived usefulness on behavioral intentions than those from urban areas. In other words, if face masks are considered as effective protection against the coronavirus, rural residents will be more likely than their urban counterparts to wear masks. Three possible reasons are provided: 1. It is well established that the rural regions are comprehensively lagging behind in terms of medical and health care services and resources compared to the urban regions in China [61], and the behavior of wearing a mask is low cost and high yield; therefore, if the rural populations note the usefulness of face masks in preventing the coronavirus, they are more likely to act. This means that public education about the usefulness of face masks in preventing the transmission of COVID-19 is more urgent in rural and remote areas in China. 2. A huge migration of young people to many urban regions in China has taken place in the past 20 years, with the elderly making up the majority of those left behind in rural areas. However, older adults in rural areas may in general be more susceptible to severe COVID-19 infections. With the potential of a higher risk of contracting COVID-19, they are more likely than younger individuals to wear masks if they perceive it is useful to prevent transmission [46]. 3. It is generally considered that rural populations engage less in preventive health behaviors than urban populations because of their lower level of information appraisal skills compared to urban residents [48]. Therefore, we may assert that the perceived usefulness of face masks indicates that the rural residents have obtained adequate information, so that they are more inclined to perform preventive health behaviors. To summarize, from a policy perspective, it is nevertheless important to take rural–urban discrepancies into account when formulating the measures taken to tackle epidemics.

The limitations of this research are threefold: first, the results of this research are constrained by the nature of the cross-sectional data, which may only have limited control over unobserved heterogeneity. Additionally, changes of behavioral intention need to be measured in different time nodes, because this would not have remained the same during the pandemic period. Second, if we consider that this pandemic may continue across the world for an indeterminate period, the results of this study could be used as an important reference for follow-up research in other countries and regions, where the attitude and intention towards mask use may be entirely different from the Chinese context. Third, this article mainly focused on online internet users; therefore, there might be data biases due to the nature of the online survey. A future study may focus on residents with limited internet access.

## Figures and Tables

**Table 1:** Demographic Characteristics.

Items	Frequency	Percentage (%)
**Gender**		
Female	742	64.9
Male	402	35.1
**Age Group (Years)**		
<18	50	4.4
19–29	509	44.5
30–39	304	26.6
40–49	163	14.2
>50	118	10.3
**Educational Attainment**		
Lower secondary and below	20	1.7
Upper secondary/Technical secondary	88	7.7
Post-secondary	102	8.9
Bachelor’s degree	612	53.5
Master’s degree	210	18.4
Doctorate degree	112	9.8
**Income (Yuan/Month)**		
<5000	616	53.8
5001–10,000	359	31.4
10,001–15,000	107	9.4
>15,001	62	5.4

**Table 2:** CFA (*n* = 1144).

Paths	Standardized Loadings (*t*-Value)	CR	AVE	Cronbach’s α
Recommended Value Perceived Usefulness 1	0.500–0.950 0.853 ***	>0.600 0.846	>0.500 0.647	>0.7000 0.821
Perceived Usefulness 2	0.739 *** (27.635)			
Perceived Usefulness 3	0.817 *** (31.486)			
Perceived Risk 1	0.748 ***	0.849	0.653	0.847
Perceived Risk 2	0.815 *** (26.304)			
Perceived Risk 3	0.858 *** (27.169)			
Social Image 1	0.913 ***	0.947	0.857	0.947
Social Image 2	0.908 *** (50.355)			
Social Image 3	0.955 *** (56.750)			
Social Pressure 1	0.739 ***	0.762	0.519	0.756
Social Pressure 2	0.624 *** (18.617)			
Social Pressure 3	0.788 *** (22.065)			
Attitude 1	0.860 ***	0.925	0.756	0.918
Attitude 2	0.897 *** (41.75)			
Attitude 3	0.931 *** (44.717)			
Attitude 4	0.781 *** (32.692)			
Behavioral Intention 1	0.878 ***	0.821	0.608	0.808
Behavioral Intention 2	0.691 *** (24.437)			
Behavioral Intention 3	0.758 *** (27.189)			

Note: *** *p* < 0.001 Model Fit: CFI = 0.948; GFI = 0.909; NFI = 0.940; SRMR = 0.056; RMSEA = 0.072. CFI: comparative fit index; GFI: goodness-of-fit index; NFI: normed fit index; SRMR: standardized root mean square residual; RMSEA: root mean square error of approximation.

**Table 3:** Model Comparison.

Models	Variables	χ2	df	GFI	CFI	RMSEA	Model Comparison	Δ2	Δdf
Model 1	U, R, I, P, A, BI	942.748	136	0.909	0.948	0.072			
Model 2	U + R, I, P, A, BI	1684.562	141	0.844	0.900	0.098	2 vs. 1	741.814 ***	5
Model 3	U + R, I + P, A, BI	2487.751	145	0.795	0.848	0.119	3 vs. 2	803.189 ***	4
Model 4	U + R + I + P, A, BI	5126.886	148	0.565	0.677	0.172	4 vs. 3	2639.135 ***	3
Model 5	U + R + I + P + A + BI	5941.412	150	0.628	0.625	0.184	5 vs. 4	814.526 ***	2

Note: *** *p* < 0.001. U stands for perceived usefulness; R stands for perceived risk; I stands for social image; P stands for social pressure; A stands for attitude; BI stands for behavioral intention. U + R indicates that the perceived usefulness and the perceived risk were combined into one factor in model 2; I + P means the social image and the social pressure were combined into one factor in model 3; U + R + I + P means the perceived usefulness, the perceived risk, the social image, and the social pressure were combined into one factor in model 4; U + R + I + P + A + BI means all factors were combined into one factor.

**Table 4:** Results of Hypothesis Testing.

Hypotheses	Paths	Path Coefficient	*p*-Value	Results
H1	Attitude → Behavioral Intention	0.442	***	Supported
H2a	Perceived Usefulness → Attitude	0.500	***	Supported
H2b	Perceived Usefulness → Behavioral Intention	0.344	*	Supported
H3a	Perceived Risk → Attitude	0.090	**	Supported
H3b	Perceived Risk → Behavioral Intention	0.007	0.942	Rejected
H4a	Social Image → Attitude	0.093	***	Supported
H4b	Social Image → Behavioral Intention	−0.002	0.897	Rejected
H5a	Social Pressure → Attitude	0.380	***	Supported
H5b	Social Pressure → Behavioral Intention	0.009	0.834	Rejected

Note: * *p* < 0.05; ** *p* < 0.01; *** *p* < 0.001.

**Table 5:** Measurement Invariance Test.

Group	Models	GFI	CFI	NFI	SRMR	RMSEA	∆χ2	Full-Metric Invariance
Type of Place of Residence	Non-restricted model	0.896	0.944	0.928	0.059	0.053	∆χ2 (8) = 18.564 *p* = 0.137	Supported
	Full-Metric invariance	0.894	0.944	0.927	0.059	0.052		

**Table 6:** Results of Multi-group Analysis.

Hypotheses	Paths	Path Coefficient (Urban)	Path Coefficient (Rural)	∆χ2	Results
H6a	Perceived Usefulness → Attitude	0.515 ***	0.498 ***	∆χ2(1) = 0.902; *p* = 0.342	Rejected
H6b	Perceived Risk → Attitude	0.109 **	−0.050(*p* = 0.534)	∆χ2(1) = 3.506; *p* = 0.061	Rejected
H6c	Social Image → Attitude	0.091 ***	0.116 *	∆χ2(1) = 0.062; *p* = 0.803	Rejected
H6d	Social Pressure → Attitude	0.335 ***	0.542 ***	∆χ2(1) = 0.363; *p* = 0.547	Rejected
H7a	Perceived Usefulness → Behavioral Intention	0.300 ***	0.587 ***	∆χ2(1) = 4.411 *	Supported
H7b	Perceived Risk → Behavioral Intention	0.041(*p* = 0.328)	−0.185(*p* = 0.063)	∆χ2(1) = 4.381 *	Rejected
H7c	Social Image → Behavioral Intention	−0.025(*p* = 0.421)	0.100(*p* = 0.120)	∆χ2(1) = 3.012 *	Rejected
H7d	Social Pressure → Behavioral Intention	−0.030(*p* = 0.510)	0.180(*p* = 0.137)	∆χ2(1) = 2.635; *p* = 0.105	Rejected
H8	Attitude → Behavioral Intention	0.472 ***	0.196(*p* = 0.120)	∆χ2(1) = 2.519; *p* = 0.112	Rejected

Note: Baseline Model Fit: CFI = 0.896; GFI = 0.944; NFI = 0.928; RMSEA = 0.053. * *p* < 0.05; ** *p* < 0.01; *** *p* < 0.001.

## Data Availability

The data presented in this study are available on request from the corresponding author.
